# Advances in nanocarbon composite materials

**DOI:** 10.3762/bjnano.9.3

**Published:** 2018-01-03

**Authors:** Sharali Malik, Arkady V Krasheninnikov, Silvia Marchesan

**Affiliations:** 1 Karlsruhe Institute of Technology, Institute of Nanotechnology, Hermann-von-Helmholtz-Platz 1, 76344 Eggenstein-Leopoldshafen, Germany; 2 Aalto University, Department of Applied Physics, P.O. Box 11100, 00076 Aalto, Finland; 3 Helmholtz-Zentrum Dresden-Rossendorf, Bautzner Landstraße 400, 01328 Dresden, Germany,; 4 University of Trieste, Department of Chemical and Pharmaceutical Sciences, Via L. Giorgieri 1, Trieste 34127, Italy

**Keywords:** nano-augmented composite materials

Materials have always been crucial to human development, to the point of being used as a reference to name specific stages of development. The first was the Stone Age, then the Bronze Age and then the Iron Age and on to their equivalents in modern times viz. the Plastic Age, the Silicon Age and the Nanomaterials Age. About 70% of all technical innovations (as estimated by the German federal government) can be attributed either directly or indirectly to the properties of the materials used – solutions are being explored on how to interface nanomaterials with other components in the macroscopic world. Therefore, we could reasonably state that we are entering the Composite Age. In particular, nanocarbons display unique properties to innovate in practically all technological sectors and branches of industry. This cutting-edge use of nano-augmented composite materials has the potential to reduce environmental pollution, to conserve resources, to save energy, and generally, to improve the quality of our lives.

Since the discovery of fullerenes over thirty years ago, there has been increasing research in the area of nanocarbon materials. Research in this field was boosted first by the discovery of carbon nanotubes and then by the advent of graphene and then expanded to the area of two-dimensional materials. This Thematic Series contains reviews and articles spanning diverse areas of research and highlights promising applications for energy transfer composites, coatings, biosensors, diagnostics, biomedicine and advanced nanocarbon materials.

Many of the contributors to this Thematic Series represent a cross-section of research subjects from participants of the European Cooperation in Science & Technology (COST) Action CA15107 “MultiComp”. COST is the longest running European framework supporting transnational cooperation amongst researchers, engineers and scholars across Europe. MultiComp is a COST Action designed to bring together theorists, experimentalists, technologists and industrialists in the field of nanocarbon materials technology and currently has over 300 participants from 33 COST countries, along with participants from Belarus, Moldova, Korea, China, Japan, Australia and New Zealand.

This Thematic Series highlights virtually all subfields of advanced nanocarbon materials research, from the longer established fields of carbon nanofibers, graphene oxide (GO) and multiwalled carbon nanotubes (MWCNTs) in composite materials, to the newer areas of nanocarbon materials for use in biomedicine and diagnostics. Energy transfer materials are also well represented with articles and reviews covering aspects of engineering, thermo-mechanical properties, photovoltaics and Li-ion battery materials.

Last but not least, we would like to thank the staff at the *Beilstein Journal of Nanotechnology*, along with all contributors and referees for making this innovative Thematic Series possible.

Sharali Malik, Arkady Krasheninnikov, Silvia Marchesan

Karlsruhe, Aalto, and Trieste, November 2017

